# Machine learning approach for the prediction of 30-day mortality in patients with sepsis-associated delirium

**DOI:** 10.1371/journal.pone.0319519

**Published:** 2025-04-09

**Authors:** Xiaoli Shen, Dongfeng Shang, Weize Sun, Shuyan Ru

**Affiliations:** 1 Department of Emergency Medicine, Huishan 3rd People’s Hospital of Wuxi City, Wuxi, China; 2 Department of Clinical Laboratory, Huishan 3rd People’s Hospital of Wuxi City, Wuxi, China; 3 Department of Critical Care, Huishan 3rd People’s Hospital of Wuxi City, Wuxi, China; Bach Mai Hospital, VIETNAM

## Abstract

This study aimed to develop models for predicting the 30-day mortality of sepsis-associated delirium (SAD) by multiple machine learning (ML) algorithms. On the whole, a cohort of 3,197 SAD patients were collected from the Medical Information Mart for Intensive Care (MIMIC)-IV database. Among them, a total of 659 (20.61%) patients died following SAD. The patients who died were about 73.00 (62.00, 82.00) years old and mostly male (56.75%). Recursive feature elimination (RFE) was used to distinguish risk factors. Subsequently, six ML algorithms including artificial neural network (NNET), gradient boosting machine (GBM), adaptive boosting (Ada), random forest (RF), eXtreme Gradient Boosting (XGB) and logistic regression (LR) were employed to establish models to predict the 30-day mortality of SAD. The performance of models was assessed via both discrimination and calibration by cross-validation with 100 resamples. Overall, 10 independent predictors, including Glasgow Coma Scale (GCS), Sequential Organ Failure Assessment (SOFA), anion gap (AG), continuous renal replacement therapy (CRRT), temperature, mean corpuscular hemoglobin concentration (MCHC), vasopressor, blood urea nitrogen (BUN), base excess (BE), and bicarbonate were identified as independent predictors for the 30-day mortality of SAD. The validation cohort demonstrated that all these six models had relatively favorable differentiation, while among them, the GBM model had the highest area under the curve (AUC) of 0.845 (95% Confidence Interval (CI): 0.816, 0.874). Furthermore, the calibration curve of these six models was close to the diagonal line in the validation sets. As for decision curve analysis, the predictive models were clinically useful as well. Based on real-world research, we developed ML models to provide personalized predictions of delirium-related mortality in sepsis patients, potentially enabling clinicians to identify high-risk SAD patients more promptly.

## Introduction

Sepsis-associated delirium (SAD) is recognized as a widespread cerebral dysfunction resulting from the systemic inflammatory response to an infection, in the absence of any central nervous system (CNS) infection [1]. In terms of epidemiology, its reported incidence rates range from 17.7 to 48% among septic patients who are admitted to the intensive care unit (ICU) [2,3]. It is reported that SAD survivors are likely to develop persistent neurocognitive impairment, such as overt dementia [4]. Significantly, the presence of delirium and coma typically signals severe brain dysfunction [5]. Unfortunately, the pathophysiology of SAD remains not well understood, but it is known to involve systemic inflammation that can impair the blood-brain barrier, further resulting in the infiltration of peripheral leukocytes into the central nervous system [6]. The resultant neuroinflammation causes a state of cholinergic failure, predisposing patients to delirium [7].

Previous studies have shown that the development of SAD is linked to extended durations of mechanical ventilation and longer ICU stays [8]. As indicated by Tokuda R et al [6], delirium by itself not only worsens short-term prognosis, but it becomes a barrier to early mobilization and physical rehabilitation. Further, all these conditions may aggravate the outcome of post-intensive care syndrome (PICS) [9]. In short, as a disease manifested as prolonged or aggravated brain dysfunction, SAD can cause a great financial burden to families in individual and societies in general, respectively. However, there are still no specific predictive models, as well as no specific early warning systems for severe SAD patients. Recently, machine learning (ML) algorithms can deal with massive, redundant, non-linear clinical information [10]. Moreover, ML algorithms are good at recognizing patterns and learning from input data to classify and predict outcomes [11]. In the field of sepsis, ML algorithms are widely used, and they also show sufficient advantages, such as predicting the occurrence of sepsis, which could trigger direct clinical action including administration of antibiotics [12]. Specifically, ML-based prediction models are capable of determining optimal antibiotic dosing without the need for daily therapeutic drug monitoring (TDM) [13]. The aim of this study was to explore potential independent predictors, and then try to develop ML models that can quantitatively estimate the probability of 30-day mortality in patients with SAD.

## Methods

### Data source

This is a retrospective study conducted on the Medical Information Mart for Intensive Care (MIMIC)-IV version 2.2 [14]. Briefly, the MIMIC-IV database included comprehensive, de-identified data of patients admitted to the ICUs at the Beth Israel Deaconess Medical Center in Boston, Massachusetts, between 2008 and 2019, including data from 383,220 admissions. Requirement for individual patient consent was waived because the study did not impact clinical care. The study was written in accordance with the REporting of studies Conducted using Observational Routinely collected health Data (RECORD) statement [15].

### Participant selection

Patients were extracted from the MIMIC-IV according to the following criteria. (1) first admitted to the ICU; (2) age ≥ 18; (3) diagnosed with sepsis; (4) ICU admission time ≥ 48 h; and (5) delirium diagnosis. The Confusion Assessment Method for ICU Patients (CAM-ICU) is used for identifying delirium in this paper [16]. More precisely, SAD in our study was defined as sepsis patients with a positive assessment of CAM-ICU and delirium assessment during their stay in ICUs. The diagnosis of sepsis was based on the Third International Consensus Definitions for Sepsis (Sepsis-3), which includes patients with documented or suspected infection and Sequential Organ Failure Assessment (SOFA) [17]. People with primary encephalopathy, chronic alcohol or drug abuse, pre-existing liver or kidney failure affecting consciousness were excluded from the study.

### Predictors of 30-day mortality in SAD patients

All research data were extracted from the MIMIC-IV by Structured query language (SQL). And they included (1) patients’demographics; (2) coexisting disorders; (3) vital signs (temperature, mean artery pressure (MAP), heart rate and respiratory rate) on the day of ICU admission; (4) laboratory measurements (such as red blood cell (RBC), white blood cell (WBC) and so on) on the day of ICU admission; (5) details of therapy (vasopressor, continuous renal replacement therapy (CRRT) and mechanical ventilation (MV)); (6) severity score including Glasgow Coma Scale (GCS) and SOFA. Multiple imputation was used to deal with missing data. Details of missing data are demonstrated in S1Table.

### Statistical analysis

Continuous variables were described using means and standard deviations for normally distributed data, or medians with interquartile ranges (IQR) for non-normally distributed data. And categorical variables were expressed as total numbers with percentages. Proportions were compared using χ² test or Fisher exact tests whereas continuous variables were compared by the t test or Wilcoxon rank sum test, as appropriate.

**Fig 1:**
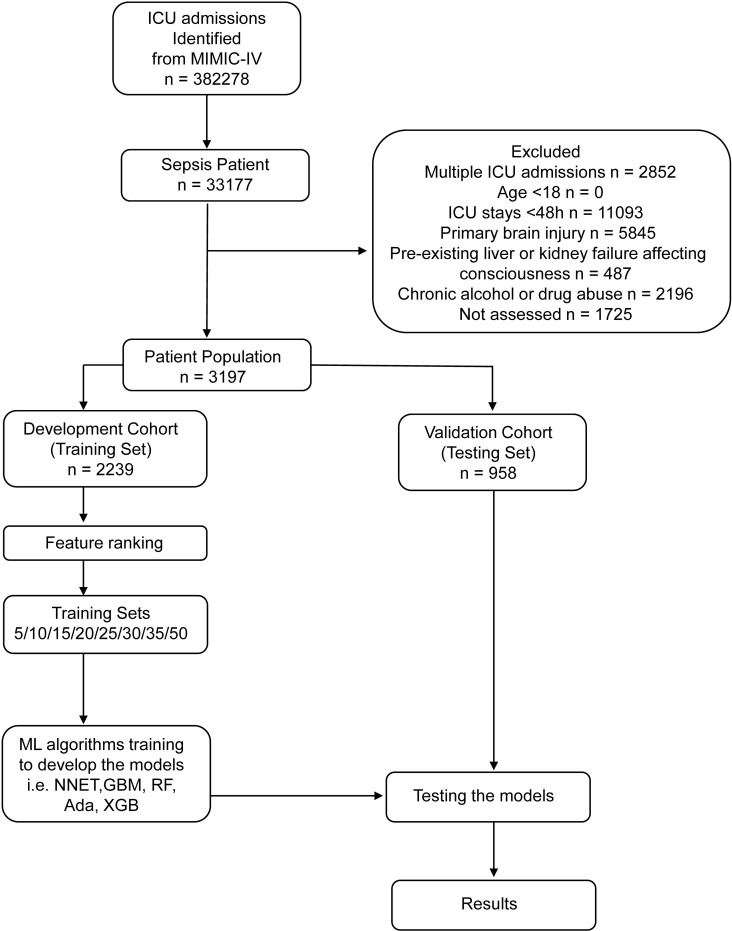
Research flowchart.

Initially, the population was randomly divided into training and validation sets in a 7:3 ratio. As a variable selection method, recursive feature elimination (RFE) was used to choose the most relevant variables related to 30-day mortality in SAD patients. In summary, RFE recursively fits into a model based on smaller resource set. In each loop, characteristics were divided by variable numbers of 5/10/15/20/25/30/35/ALL (ALL =  50 variables, as represented in Fig 1). 5-fold cross-validation was used as the resampling method to find the optimal hyperparameters. The training test process was repeated 100 times. In this study, six different ML algorithms were used to develop models, including artificial neural network (NNET), gradient boosting machine (GBM), adaptive boosting (Ada), random forest (RF), eXtreme Gradient Boosting (XGB) and logistic regression (LR). The NNET algorithm is a ML algorithm based on biological networks. Based on the backpropagation algorithm, this model relies on nodes and connections to make complex decisions [18]. GBM is a type of ML algorithm that sequentially combines weak models into a strong model. Model integration means constructing a prediction model by combining multiple base models, and the prediction performance of the final integrated model is better than that of any single base model [19]. Ada is a simple and general ensemble algorithm that creates a strong classifier by using many weak classifiers. The algorithm starts with weak learners and assigns weights to each training sample. Finally, the predictions are combined with an ensemble weight to obtain the final prediction [20]. RF is a common ML method based on decision trees and has been used in a variety of medical studies. It can be applied to various complex data sets. Through the bootstrap aggregation process, the variance of the model can be reduced, thereby improving the prediction accuracy [21]. XGB is an integrated gradient boosting method, which uses the negative gradient of the loss function as the residual value for model fitting to achieve accurate classification results [22].

Model performance was evaluated by area under the curve (AUC), accuracy, sensitivity, specificity, negative predictive value (NPV) and positive predictive value (PPV). Additionally, the calibration curve was used to evaluate how well the observed outcomes matched the predicted outcomes, while the decision curve analysis assessed the net clinical benefit. The SHapley Additive exPlanations (SHAP) method was applied to interpret the final predictive model. Higher SHAP values indicated a greater predicted probability of mortality among SAD patients [23]. In order to facilitate clinical use, we also launched a web page by “Shiny” application. For missing values, the method of multiple imputation was used.

All analyses were conducted using R version 4.0.2 (http://www.R-project.org, The R Foundation). For our study, we utilized the “Caret” and “Shiny” R packages to facilitate the process. Statistical significance was defined as *P* values of less than 0.05 (two-sided test).

## Results

### Participants and baseline characteristics

Based on the inclusion and exclusion criteria, the final dataset comprised 3,197 patients. Fig 1 demonstrated the process of data extraction, training preparation, data testing by multiple ML algorithms. Baseline characteristics of all included patients are shown in Table 1. Dead individuals were generally older and had more comorbidities, including myocardial infarction, mild and severe liver disease, renal disease, malignant cancer, and metastatic solid tumors. They also exhibited higher values for the Charlson Comorbidity Index (CCI), as well as elevated heart rate, respiratory rate, white blood cell count (WBC), red blood cell volume distribution width (RDW), activated partial thromboplastin time (APTT), prothrombin time (PT), international normalized ratio (INR), lactate, base excess (BE), anion gap (AG), potassium, glucose, creatinine (CRE), and blood urea nitrogen (BUN). As for therapy, SAD people who died were more likely to receive vasopressor, CRRT, and MV. Disease severity score indicated that lower GCS and higher SOFA occurred in SAD patients who died. Additionally, these individuals typically had lower temperature, mean arterial pressure (MAP), RBC, mean corpuscular hemoglobin concentration (MCHC), platelet count (PLT), hematocrit (HCT), pH, bicarbonate, partial pressure of oxygen (PaO_2_), chloride, and calcium levels. In summary, patients who died had a poorer baseline health status.

**Table 1:** Baseline characteristic variables.

Variables	Survival (n = 2538)	Died (n = 659)	*P* Value
**Demographics**			
Median age (IQR), y	70.00 (59.00, 81.00)	73.00 (62.00, 82.00)	0.001
Male, n (%)	1378 (54.29)	374 (56.75)	0.278
Race, n (%)			0.123
Black	62 (2.61)	23 (4.28)	
White	226 (9.52)	43 (8.01)	
Hispanic	92 (3.87)	15 (2.79)	
Asian	1914 (80.59)	434 (80.82)	
Others	81 (3.41)	22 (4.10)	
**Coexisting disorders, n (%)**			
Myocardial infarction	421 (16.59)	168 (25.49)	<0.001
Congestive heart failure	935 (36.84)	264 (40.06)	0.140
Peripheral vascular disease	310 (12.21)	88 (13.35)	0.470
Cerebrovascular disease	88 (3.47)	17 (2.58)	0.390
chronic pulmonary disease	794 (31.28)	217 (32.93)	0.446
Rheumatic disease	76 (2.99)	23 (3.49)	0.597
Peptic ulcer disease	58 (2.29)	20 (3.03)	0.332
Mild liver disease	246 (9.69)	144 (21.85)	<0.001
Diabetes without complication	699 (27.54)	162 (24.58)	0.140
Diabetes with complication	203 (8.00)	53 (8.04)	1.000
Paraplegia	45 (1.77)	6 (0.91)	0.161
Renal disease	594 (23.40)	197 (29.89)	0.001
Malignant cancer	334 (13.16)	128 (19.42)	<0.001
Severe liver disease	77 (3.03)	46 (6.98)	<0.001
Metastatic solid tumor	157 (6.19)	93 (14.11)	<0.001
AIDS	30 (1.18)	2 (0.30)	0.072
CCI, median (IQR)	6.00 (4.00, 8.00)	7.00 (5.00, 9.00)	<0.001
**Vital signs on the day of ICU admission**			
Temperature (°C), median (IQR)	36.80 (36.40, 37.30)	36.60 (36.10, 37.10)	<0.001
MAP (mmHg), median (IQR)	74.00 (69.00, 81.00)	73.00 (67.00, 79.00)	<0.001
Heart rate (/min), median (IQR)	86.00 (77.00, 99.00)	90.00 (78.00, 104.00)	<0.001
Respiratory rate (/min), median (IQR)	19.00 (17.00, 23.00)	22.00 (19.00, 25.00)	<0.001
**Laboratory findings on the day of ICU admission**			
RBC (×10^9^/L), median (IQR)	3.50 (3.10, 3.90)	3.40 (3.00, 4.00)	0.245
WBC (×10^9^/L), median (IQR)	11.70 (8.50, 15.80)	13.70 (9.25, 19.00)	<0.001
MCH (PG), median (IQR)	30.10 (28.70, 31.50)	30.30 (28.65, 31.60)	0.648
MCHC (%), median (IQR)	33.50 (32.40, 34.40)	32.60 (31.50, 33.70)	<0.001
Platelet (×10^9^/L), median (IQR)	204.00 (147.00, 275.62)	186.00 (118.25, 269.00)	<0.001
RDW (%), median (IQR)	14.90 (14.00, 16.30)	15.80 (14.40, 17.85)	<0.001
Hematocrit (%), median (IQR)	30.70 (27.80, 34.40)	30.70 (27.60, 35.70)	0.211
APTT (seconds), median (IQR)	32.80 (27.80, 40.90)	35.70 (29.50, 49.30)	<0.001
PT (s), median (IQR)	14.70 (13.50, 16.60)	16.10 (13.60, 20.90)	<0.001
INR, median (IQR)	1.30 (1.20, 1.50)	1.50 (1.20, 1.90)	<0.001
PH, median (IQR)	7.40 (7.30, 7.40)	7.30 (7.30, 7.40)	<0.001
Bicarbonate (mmol/L), median (IQR)	23.70 (21.00, 26.50)	21.00 (17.80, 24.70)	<0.001
Lactate (mmol/L), median (IQR)	1.70 (1.20, 2.40)	2.60 (1.52, 4.27)	<0.001
BE (mEq/L), median (IQR)	-0.40 (-3.20, 1.50)	-3.30 (-7.00, 0.00)	<0.001
Anion gap (mmol/L), median (IQR)	13.00 (11.30, 15.50)	16.00 (13.30, 19.20)	<0.001
Chloride (mmol/L), median (IQR)	106.00 (101.70, 109.50)	104.30 (99.60, 108.75)	<0.001
Calcium (mmol/L), median (IQR)	8.20 (7.70, 8.60)	8.10 (7.60, 8.53)	0.001
Sodium (mmol/L), median (IQR)	138.80 (136.12, 141.00)	138.50 (135.00, 142.00)	0.648
Potassium (mmol/L), median (IQR)	4.20 (3.90, 4.60)	4.30 (3.90, 4.80)	<0.001
Glucose (mmol/L), median (IQR)	129.30 (108.75, 161.00)	141.00 (109.50, 185.65)	<0.001
CRE (mg/dl), median (IQR)	1.10 (0.80, 1.60)	1.50 (1.00, 2.50)	<0.001
BUN (mg/dl), median (IQR)	21.70 (14.80, 36.80)	32.80 (21.05, 51.85)	<0.001
**Therapy (1st 24h), n (%)**			
Vasopressor	1333 (52.52)	510 (77.39)	<0.001
CRRT	101 (3.98)	124 (18.82)	<0.001
MV	2426 (95.59)	650 (98.63)	<0.001
**Scoring system**			
GCS	13.00 (9.00, 14.00)	8.00 (3.00, 14.00)	<0.001
SOFA	6.00 (4.00, 8.00)	10.00 (7.00, 13.00)	<0.001

### Variable selection

The RFE algorithm selected a total of 10 important predictors (S1 Fig). Specifically, predictors included GCS, SOFA, AG, CRRT, temperature, MCHC, vasopressor, BUN, BE, and bicarbonate. Subsequently, these 10 variables were used in all analysis for all models in both training and testing sets.

### Model performance

**Fig 2:**
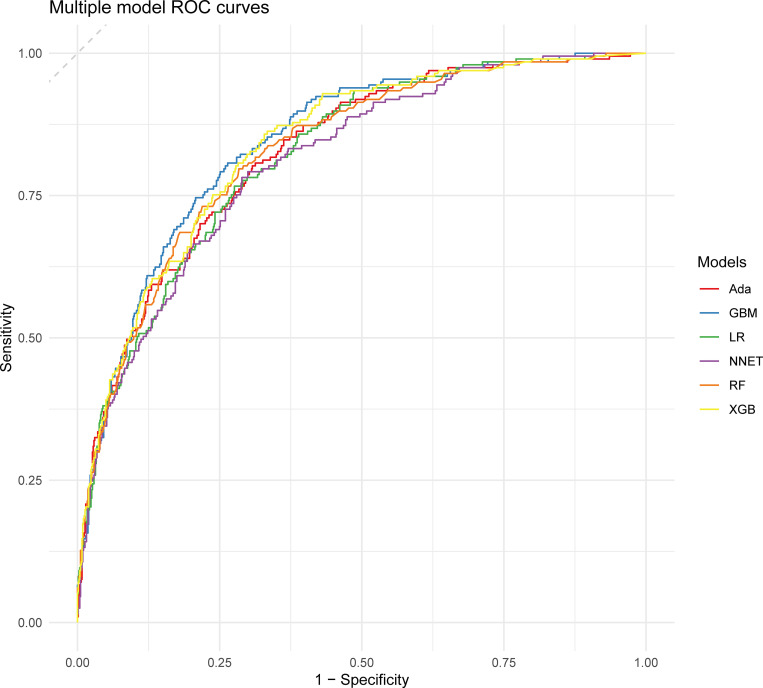
Area under the curve of receiver operating characteristic curve by machine learning models in the validation cohort.

**Fig 3:**
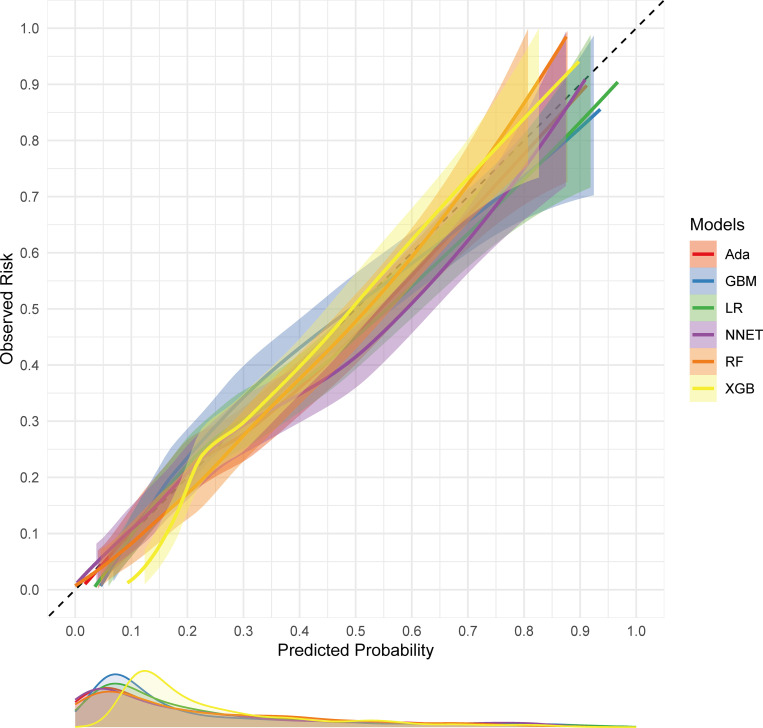
Calibration curve in the validation cohort.

**Fig 4:**
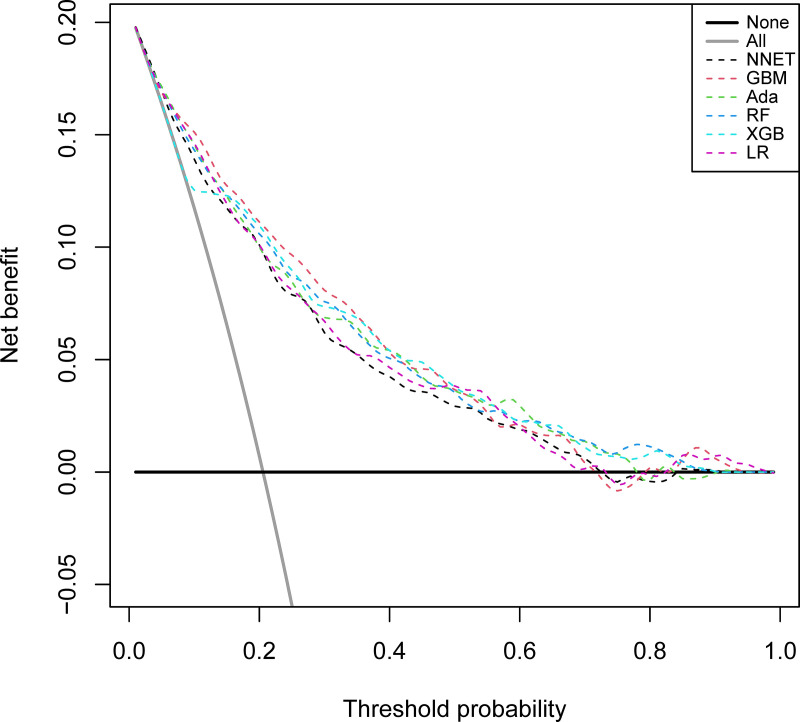
Decision curve analysis in the prediction of SAD mortality.

Table 2 and Fig 2 describe the predictive performance of these models on the validation set. By comparison, GBM had the highest predictive performance among these six models (AUC 0.845, 95% confidence interval (CI) 0.816 to 0.874). As for the calibration performance, the NNET, GBM, Ada, RF and LR models had a good calibration—namely, being close to the ideal diagonal line, with P-values of 0.052, 0.345, 0.632, 0.356 and 0.346, respectively (Hosmer-Lemeshow test) (Fig 3). Furthermore, decision curve analysis (DCA) was performed on these three models, and the results are shown in Fig 4. The analysis indicated that these four prediction models provided favorable net benefit for predicting 30-day mortality of SAD. To be specific, the X-axis indicated the threshold probability for critical care outcome and Y-axis indicated the net benefit. (red line =  Ada, blue line =  GBM, green line =  LR, purple line = NNET, orange line =  RF, yellow line =  XGB).

**Table 2:** Analysis of sensitivity and specificity.

Model	Accuracy	Sensitivity	Specificity	PPV	NPV	Operating threshold	AUC	95% CI
NNET	0.725	0.782	0.711	0.412	0.926	0.197	0.810	(0.777, 0.842)
GBM	0.751	0.807	0.736	0.442	0.936	0.173	0.845	(0.816, 0.874)
Ada	0.715	0.802	0.693	0.403	0.931	0.183	0.826	(0.795, 0.857)
RF	0.733	0.716	0.797	0.421	0.932	0.218	0.829	(0.797, 0.860)
XGB	0.710	0.858	0.671	0.403	0.948	0.198	0.837	(0.807, 0.867)
LR	0.733	0.766	0.724	0.418	0.923	0.199	0.821	(0.790,0.852)

PPV, positive predictive values; NPV, negative predictive values, AUC, area under the curve; CI, confidence interval; NNET, artificial neural network; GBM, gradient boosting machine; Ada, Adaptive boosting; RF, random forest; XGB, eXtreme Gradient Boosting; LR, logistic regression.

### Model interpretations

**Fig 5:**
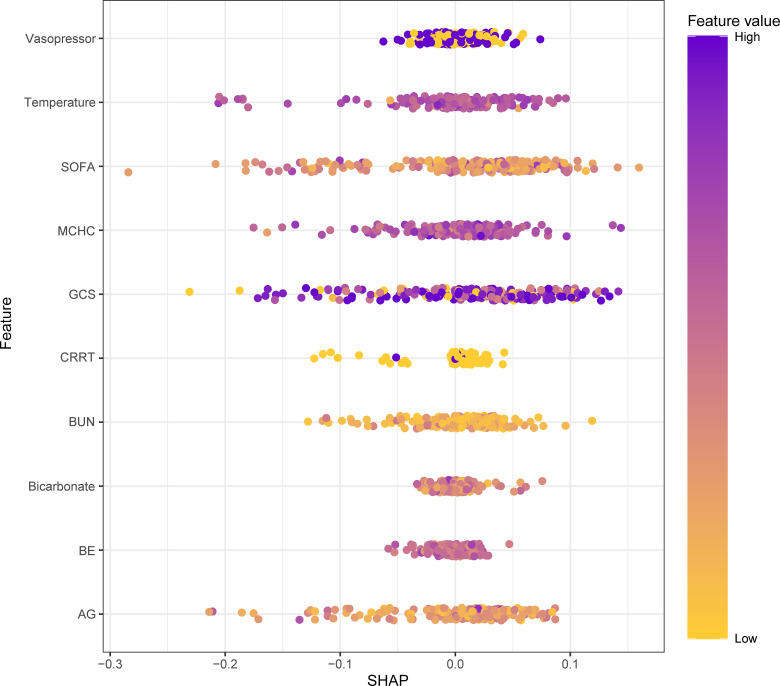
SHAP summary plot of the GBM model (top 10 features).

To determine the key factors in the model, we visualized the feature importance ranking using the GBM algorithm. As shown in Fig 5, each variable included in the study showed different levels of significance for SAD mortality based on the GBM approach. Overall, GCS emerged as the most crucial factor, followed by MCHC, temperature, and so forth.

### Model application

**Fig 6:**
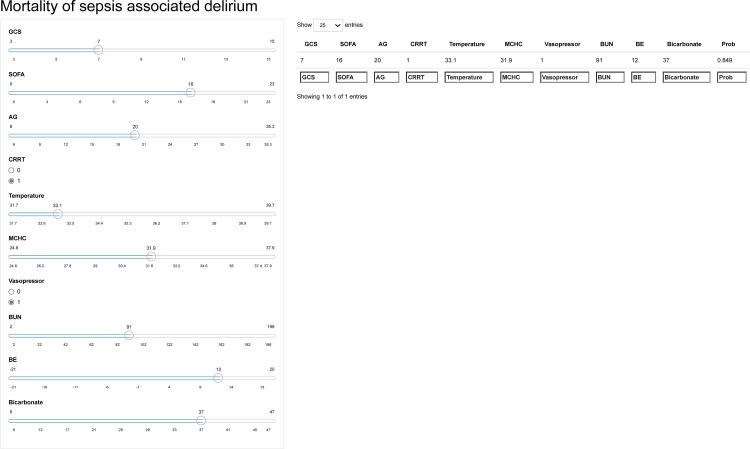
Examples of website usage. Entering the input value determined the mortality and displayed how each value contributed to the prediction.

In order to further visualize the results, and facilitate the use of GBM models by clinicians, we further uploaded an online calculator (https://pcstudy.shinyapps.io/Mortality_of_sad/). As shown in Fig 6, the Shiny package further visualizes the results, explaining the weight of each variable in predicting 30-day mortality for SAD. Take a patient for example: GCS (7), SOFA (16), AG (20), CRRT, temperature (33.1 °C), MCHC (31.9), vasopressor, BUN (91), BE (12), bicarbonate (37). Through model analysis, the 30-day mortality risk of SAD patients was 84.90%, indicating that the 30-day mortality rate of SAD patients was high.

## Discussion

Timely and swift recognition of SAD patients who have a poor prognosis continues to be a significant challenge. In this research, our findings indicated that around 20.61% of SAD patients in the ICU succumbed to their condition, with SAD patients in the mortality group having older ages, more comorbidities, worse vital signs compared to those in the non-mortality group. We then used RFE to screen variables most associated with death. Then, six models including NNET, GBM, Ada, RF, XGB and LR, based on ML algorithms were established, Comparably, GBM-based ML predictive model demonstrated commendable predictive performance in the validation cohort, enabling early prediction of the 30-day mortality of SAD on ICU admission. For ease of use by clinicians, we then developed a web-based calculator.

Existing research on SAD has focused on the diagnosis of SAD or examined incidence and risk factors. For instance, Gu Q et al [24] found that SOFA score, requiring mechanical ventilation, first lactate level, and first serum phosphate on ICU admission are indicators that can forecast the onset of delirium in sepsis patients. Another study suggested that emergency surgery, higher doses of midazolam, and fentanyl could serve as independent risk factors for SAD in mechanically ventilated patients suffering from sepsis [3]. In addition, in Zhang Y’s study [25], the XGBoost model outperformed other models in predicting SAD. It is worth mentioning that these studies have focused on the diagnosis of SAD, and few studies have predicted the prognosis of SAD. Previous study conducted by Zhang L et al [26] identified five independent risk factors for developing a prediction model for mortality in SAD: total length of stay in the ICU, hemoglobin level, albumin level, activated partial thromboplastin time, and total bilirubin level. But simple logistic regression may not fit the relationship between variables well. Actually, predictions about mortality can help doctors identify patients with critical SAD. In this study, ten variables were identified as predictors, involving GCS, SOFA, AG, CRRT, temperature, MCHC, vasopressor, BUN, BE, and bicarbonate. Research involving 16,343 sepsis patients revealed that the initial 24-hour GCS score is a significant predictor of SAD, aligning with the findings of two earlier models for diagnosing SAD [25,27,28]. Apart from that, Peng L [29] reported that systematic scores including GCS and SOFA can be used as predictors for the prognosis of SAD patients. Additionally, a series of previous studies also have found that higher values of the GCS and SOFA were related to the mortality rate of SAD patients [6,30,31]. As an accepted method of assessing the severity of a patient’s condition upon admission to the ICU, GCS and SOFA are tools to forecast the results for patients in the ICU [32]. In our study, we found that GCS and SOFA were prominent variables in the importance plot, indicating their significant predictive capability for the 30-day mortality of SAD patients. This highlights the utilization of the GCS and SOFA in evaluating the severity and prognosis of individuals suffering from SAD. Patients with SAD who experience multiple organ dysfunction have a higher likelihood of mortality. Cascade immune response, circulatory abnormalities, coupled with metabolic acidosis increase may be associated with such a complicated pathophysiological process[33]. Thus, the management of SAD mainly concerns the treatment of sepsis itself and the correction of potential neurotoxic dysfunction. Moreover, our study found that body temperature among vital signs was associated with the prognosis of SAD. The potential mechanism may be that inflammatory responses caused by temperature can disrupt thermoregulatory mechanisms, resulting in fever or hypothermia. Sustained abnormal body temperature can disrupt blood-brain barrier and neural function during SAD [30,34]. Jin Z et al [35] found that temperature control has some association with the risk of postoperative delirium. Similarly, in Ju JW et al’s study [36], intraoperative hypothermia was significantly associated with an increased risk of postoperative delirium.

In clinical practice, vasopressin is increasingly utilized as a vasoactive agent. A prior study by Maheshwari et al discovered a significant association between changes in blood pressure and decreased survival in patients with septic shock [37]. Early-stage resuscitation is generally recognized as a promising approach for treating sepsis, and administering vasopressin can reduce the severity of the condition, potentially aiding in cognitive improvement for patients with SAD [38, 39]. Additionally, patients with septic shock need vasoactive agents to ensure sufficient tissue perfusion. Following norepinephrine, vasopressin is recommended as a second-line adjunctive treatment for those with ongoing insufficient mean arterial pressure [40].

Additionally, Chen J et al [41] found that low MCHC was risk factors of delirium, which was similar to our findings. During sepsis, systemic inflammation and oxidative stress can disrupt erythropoiesis by lowering erythropoietin (EPO) levels and reducing iron availability, which subsequently leads to decreased hemoglobin levels [42–45]. Worse all, inflammation can interfere with the maturation of RBCs in the bone marrow, causing the premature release of immature RBCs into the bloodstream, further disturbing the hemoglobin level [46, 47]. Moreover, decreased MCHC leads to reduced oxygen transport capacity and hypoxia of tissue cells, and MCHC is also a marker of malnutrition [48]. Therefore, tracking hemoglobin levels is essential for managing sepsis and assessing long-term survival prospects in patients with SAD.

The study’s strengths are found in its use of ML. Based on parameters collected within 24 hours of admission in ICU, this ML model is able to predict the mortality of SAD much earlier. In addition, cross-validation can decrease potential overfitting.

There were limitations in this study. First, as a retrospective observational study, it has inherent limitations, such as the potential presence of unmeasured confounders. Second, since the study was conducted at a single center, further validation is needed to ensure its findings are applicable to other populations. Third, ML algorithms are susceptible to data biases, such as the selection of specific patients, and potential biases inherent in retrospective datasets [49,50]. Fourth, the prediction process of ML algorithm is mostly “black box”, and its complex process makes many clinicians remain wary of the model. Thus, there has been a recent explosion of research in the field of explainable ML aimed at addressing these concerns [51]. Lastly, the ML algorithm is dependent on the data set used for training, and its universal application may be limited by different clinical environments.

## Conclusion

In summary, SAD is prevalent among ICU patients with sepsis and is associated with higher mortality rates compared to sepsis alone. By employing a ML-based early prediction model, we can more effectively predict 30-day mortality in SAD patients. Moreover, developing this model aids in early risk detection and allows for the implementation of preventive strategies, which could help lower the incidence and mortality of SAD to some degree.

## Supporting information

S1 Table Missing number (%) for included variables in the dataset. (DOCX)

S1 Fig Variable selection by RFE. (TIFF)
