# Supplementary material for: Machine learning approach for the prediction of 30-day mortality in patients with sepsis-associated delirium
Source: PLoS One. 2025 Apr 9;20(4):e0319519. doi: 10.1371/journal.pone.0319519 (PMC11981165; doi:10.1371/journal.pone.0319519)
Supplement: S1 Table — (DOCX) [file pone.0319519.s001.docx]

| Table S1 Missing number (%) for included variables in the dataset | |
| --- | --- |
| Variables | Missing, N (%) |
| Race | 8.91 |
| Temperature | 7.13 |
| MAP | 0.44 |
| Heart rate | 0.44 |
| Respiratory rate | 0.44 |
| RBC | 0.06 |
| WBC | 0.09 |
| MCH | 0.06 |
| MCHC | 0.06 |
| Platelet | 0.06 |
| RDW | 0.06 |
| APTT | 5.72 |
| PT | 5.60 |
| INR | 5.60 |
| Hematocrit | 0.03 |
| PH | 16.77 |
| BE | 16.77 |
| Anion gap | 0.47 |
| Calcium | 10.32 |
| Sodium | 0.13 |
| Potassium | 0.29 |
| Glucose | 0.75 |
| BUN | 0.06 |
| MAP, mean artery pressure; RBC, red blood cell; WBC, white blood cell; MCH, mean corpuscular hemoglobin; MCHC, mean corpuscular hemoglobin concentration; RDW, red blood cell volume distribution width; APTT, activated partial thromboplastin time; PT, prothrombin time; INR, international normalized ratio; PH, potential of hydrogen; BE, buffer excess; BUN, blood urea nitrogen; | |
